# A study of genetic association with electrophysiological measures related to alcoholism: GAW14 data

**DOI:** 10.1186/1471-2156-6-S1-S126

**Published:** 2005-12-30

**Authors:** Ao Yuan, Victor Apprey, Jules P Harrell, Robert E Taylor, George E Bonney

**Affiliations:** 1 National Human Genome Center, Howard University, Washington DC, USA; 2 Department of Community Health and Family Medicine, Howard University, Washington DC, USA; 3 Department of Psychology, Howard University, Washington DC, USA; 4 Division of Medical Genetics, Department of Pediatrics, Howard University, Washington DC, USA; 5 Alcoholism Research Center, Howard University, Washington DC, USA

## Abstract

Recently, alcohol-related traits have been shown to have a genetic component. Here, we study the association of specific genetic measures in one of the three sets of electrophysiological measures in families with alcoholism distributed as part of the Genetic Analysis Workshop 14 data, the NTTH (non-target case of Visual Oddball experiment for 4 electrode placements) phenotypes: ntth1, ntth2, ntth3, and ntth4. We focused on the analysis of the 786 Affymetrix markers on chromosome 4. Our desire was to find at least a partial answer to the question of whether ntth1, ntth2, ntth3, and ntth4 are separately or jointly genetically controlled, so we studied the principal components that explain most of the covariation of the four quantitative traits. The first principal component, which explains 70% of the covariation, showed association but not genetic linkage to two markers: tsc0272102 and tsc0560854. On the other hand, ntth1 appeared to be the trait driving the variation in the second principal component, which showed association and genetic linkage at markers in four regions: tsc0045058, tsc1213381, tsc0055068, and tsc0051777 at map distances 53.26, 85.42, 89.31, and 172.86, respectively. These results show that the partial answer to our starting question for this brief analysis is that the NTTH phenotypes are not jointly genetically controlled. The component ntth1 displays marked genetic linkage.

## Background

Recently, evidence has been found to relate alcoholism to genetic factors [1-4]. The Collaborative Study on the Genetics of Alcoholism (COGA) is a program to study this phenomenon extensively. For the Genetic Analysis Workshop 14 (GAW14), an expanded dataset was released for analysis. It contains multiple phenotypes and genome-wide scans. We chose to study genetic association of electrophysiological measures related to alcoholism focusing on the NTTH phenotypes and the 786 Affymetrix single-nucleotide polymorphisms (SNPs) on chromosome 4. This chromosome has been shown to be involved in NTTH phenotypes in some previous studies.

There are four NTTH phenotypes: ntth1, ntth2, ntth3, and ntth4. For the four correlated traits a natural question is, are they mediated by the same set of genes, or, is each separately genetically controlled? Here we attempt to find a partial answer to these questions.

## Methods

In view of our desire to determine whether ntth1, ntth2, ntth3, and ntth4 are separately or jointly genetically controlled, we shall study the principal components that explain most of the covariation of the four quantitative traits. We shall then analyze the promising components separately.

For the association and linkage analyses, we used our Genetic Epidemiology Models (GEMs) package, which has routines for the regressive models for quantitative traits [5,6]. Typically for this problem, one may perform a linkage analysis to pinpoint the highly spurious region, but this is computationally intensive and time consuming. In this dataset, the number of SNPs is large. For chromosome 4 alone there are 786 SNPs. We did a two-stage analysis. The first stage is an association analysis in which we regressed the trait on age, sex, and the SNPs, one at a time, across all the 786 SNPs on chromosome 4. Strong statistical association between the phenotypes and the SNPs provided us the basis to select the phenotypes/SNPs for the next stage of analysis. In the second stage, a formal linkage analysis was performed on the SNPs selected from the first stage.

## Results

The proportion of the total variance explained by the first, second, third and fourth components are: 0.698, 0.195, 0.084, and 0.024, respectively.

The factor loadings for the first and second principal components are listed in Table 1. The first loading shows a rather even spread, and along with the amount of covariance explained by principal components, explains almost 70% of the covariation among the four traits. The loadings for the second component, which explains only 19.5% of the covariation, are quite illuminating. The relatively huge loading of 0.930 for ntth1 suggests that ntth1 itself rather than the combination with the others is the important trait. So we first analyzed the first principal component, which explains almost 70% of the covariation, i.e.,

0.338ntth1 + 0.515ntth2 + 0.569ntth3 + 0.545ntth4.

Instead of analyzing the second principal component, which, explains 20% of the covariation, we analyzed ntth1, which is the driving factor. The third and fourth principal components account for little covariation so they were not analyzed. However, we also performed univariate linkage analysis on ntth2, ntth3, and ntth4 for comparison.

**Figure 1:**
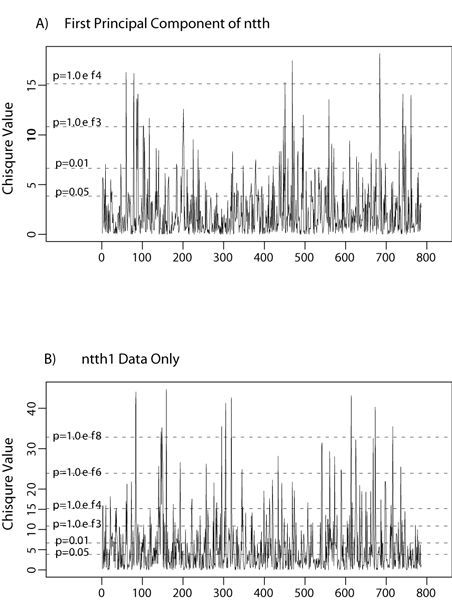
ntth chi-square values for chromosome 4 SNPs.

The results of the association study for the first principal component are summarized in Figure 1A, which displays the values of chi-square ratios for association versus no association for each SNP. The four horizontal lines indicate the significant levels. We find that at the 0.0001 level there are five SNPs showing significant associations. These five sit roughly around locations 60, 70, 450, 470, and 690.

The association analysis results for ntth1 alone are displayed in Figure 1B. We see ten SNPs significant at the 10^-8 ^level. This is a far stronger result than those using the principal component combinations. Although Figures 1A and 1B show many peaks, the remarkable difference is that the markers around position 300 are prominent in ntth1 but not in the first principal component.

Table 2 presents the linkage analysis results for those SNPs that showed significant association at the 0.00001 level in the first stage of analysis. The very low level of significance was chosen because of concerns about multiple comparison. The LOD scores and chi-square values in Table 2 are from the second stage of linkage analysis. The marker tsc0560854 at position 469 had significant chi-square value at the 5% level, but it is not significant by the conventional linkage standard of LOD score 2.0 or higher. The results suggest that the principal components method is not adequate for this problem. It means that using the first principal linear combination of the phenotypes may have smoothed/filtered out the linkage effect.

The linkage results for ntth1 alone are presented in Table 3. It is interesting to note that the marker tsc0560854 at position 469, which showed significant chi-square value in the first principal component, were not significant for ntth1 and so are not in Table 3. On the other hand, markers tsc0045058 at position 148, tsc1213381 at position 295, tsc0055068 at position 319, and tsc0051777 at position 714 showed significant linkage; these markers were at map distances 53.26, 85.42, 89.31, and 172.86, respectively. For ntth1, other significant linkage LOD score peaks occur at tsc1276837 (map distance 34.24), tsc0526379 (map distance 52.99), and at tsc0527513 (map distance 55.57). Also, there are some SNPs showing significant association in Figure 1B, but not all of them showing evidence of linkage as in Table 3. We think the significant association in the first stage of analysis indicate some non-random association between the phenotype and the SNPs, which may suggest Hardy-Weinberg disequilibrium among the SNPs or somthing else, not necessarily linkage.

The linkage analysis results for ntth2, ntth3, and ntth4 are presented in Table 4, showing no linkage to the selected markers that showed significant association with the trait in the first stage of analysis.

## Conclusion

Our analysis of the four NTTH phenotypes, although brief, is revealing. The first principal component, which explains 70% of the covariation, showed association but not linkage to two markers: tsc0272102 and tsc0560854. On the other hand, ntth1, which was the trait driving the variation in the second principal component, showed association and linkage at markers in four regions: tsc0045058, tsc1213381, tsc0055068, and tsc0051777 at map distances 53.26, 85.42, 89.31, and 172.86 respectively.

## Abbreviations

COGA: Collaborative Study on the Genetics of Alcoholism

GAW14: Genetic Analysis Workshop 14

SNP: Single-nucleotide polymorphism

## Authors' contributions

AY worked on the methodology, participated in the computations, and drafted the manuscript. VA implemented the computer program and participated in the computations. JPH participated in the analysis. RET participated in the analysis and interpretation of results. GEB helped to decide on the methodology, participated in the analysis, and assisted in writing the manuscript.

## Figures and Tables

**Table 1:** Factor loadings for first two principal components

	ntth1	ntth2	ntth3	ntth4
1^st ^component	0.338	0.515	0.569	0.545
2^nd ^component	0.930	0.294	0.200	0.000

**Table 2:** Linkage results on chromosome 4 for the first principal component of ntth

SNP	Marker name	Map distance	Allele	LOD Score	Chi-square value
60	tsc0273260	28.64	1	0.00	0.00
79	tsc0272102	32.91	1	0.44	2.03
451	tsc0538170	120.48	1	0.00	0.00
469	tsc0560854	122.85	1	0.85	3.93
684	tsc0047209	166.94	1	0.00	0.00

**Table 3:** Linkage results on chromosome 4 for ntth1

SNP	Marker name	Map distance	Allele	LOD score	Chi-square value
84	tsc1276837	34.24	2	1.75	8.05
146	tsc0526379	52.99	1	1.92	8.87
148	tsc0045058	53.26	1	3.35	15.45
159	tsc0527513	55.57	2	1.93	8.89
295	tsc1213381	85.42	2	2.22	10.25
305	tsc0744013	87.05	2	0.75	3.48
319	tsc0055068	89.31	2	3.03	13.97
614	tsc0044851	150.40	2	0.43	1.97
673	tsc0572265	165.77	1	0.00	0.00
714	tsc0051777	172.86	2	2.57	11.83

**Table 4:** Linkage results on chromosome 4 for ntth2-ntth4

	SNP	Marker name	Map distance	Allele	LOD score	Chi-square value
ntth2	88	tsc0750487	34.6124	1	0.00	0.00
	89	tsc0568024	34.6135	1	0.00	0.00
ntth3	732	tsc0540150	177.197	1	0.00	0.00
ntth4	89	tsc0568024	34.6135	1	0.00	0.00
	732	tsc0540150	177.197	1	0.05	0.21
